# Sexual Health Behaviors and Outcomes Among Middle‐Aged and Older Disabled Adults in Britain

**DOI:** 10.1111/psrh.70034

**Published:** 2025-09-04

**Authors:** Yoshiko Sakuma, Bernice Lin, Eneyi Kpokiri, Junead Khan, Huachun Zou, Joseph D. Tucker, Tom Shakespeare, Hannah Kuper, Dan Wu

**Affiliations:** ^1^ Department of Clinical Research, Faculty of Infectious and Tropic Diseases London School of Hygiene and Tropical Medicine London UK; ^2^ MSc Epidemiology London School of Hygiene and Tropical Medicine London UK; ^3^ School of Public Health (Shenzhen) Sun Yat‐Sen University Shenzhen Guangdong China; ^4^ Institute for Infectious Diseases and Global Health University of North Carolina at Chapel Hill Chapel Hill North Carolina USA; ^5^ Department of Population Health, Faculty of Epidemiology and Population Health London School of Hygiene and Tropical Medicine London UK; ^6^ Department of Social Medicine and Health Education School of Public Health of Nanjing Medical University Nanjing China

## Abstract

**Background:**

Sexual health is crucial for well‐being, yet research often overlooks middle‐aged and older adults, as well as those with disabilities. This study explores the sexual health of disabled middle‐aged and older adults in Britain.

**Objective:**

We aim to explain sexual behaviors and outcomes among disabled adults aged 45–74 in Britain.

**Methods:**

We conducted a secondary quantitative analysis using data from the third National Survey of Sexual Attitudes and Lifestyles (Natsal‐3), a British population‐representative survey. The study did not focus on a specific type of disability but rather included people who consider themselves to have a long‐standing illness that limits their activity. The analysis incorporated variables on sexual behaviors and outcomes: bivariate analyses and multiple logistic regressions stratified by sex compared behaviors and outcomes by disability status.

**Results:**

Of 7082 participants included, 1906 were classified as being with limiting disability status. Adjusting for sociodemographic differences, compared to nondisabled individuals, those with limiting disability status adults were less likely to engage in sex in the last 4 weeks (aOR: 0.60, CI: 0.51–0.71) or be in a steady relationship (aOR: 0.69, CI: 0.59–0.80) and were more likely to report poorer sexual health outcomes, including having experienced coerced sex attempts (aOR:1.83, CI:1.48–2.27), had coerced sex (aOR: 1.64, CI:1.33–2.01), had their sex life affected by health in the last year (aOR: 5.08, CI:4.27–6.05), and sought help for their sex lives (aOR:1.73, CI:1.38–2.17).

**Conclusions:**

Middle‐aged and older disabled adults in Britain are less likely to be sexually active, yet more likely to experience negative sexual health outcomes. The increased health‐seeking behaviors and their vulnerability highlight the necessity for tailored sexual health services, extending into middle age and older adulthood.

## 1 Introduction

Sexual health is crucial for fundamental well‐being and physical and mental health throughout life [1]. Respecting and protecting the sexual rights of all human beings, regardless of their age, health, and reproductive status, is essential for achieving and maintaining sexual health [2]. However, much sexual health research, interventions, and policy recommendations have focused on younger populations [3, 4], leaving groups such as middle‐aged and older adults, and individuals with disabilities, underrepresented in the field of sexual health [5, 6, 7, 8, 9]. Social misconceptions that middle‐aged and older adults, as well as disabled individuals, are “asexual” have led to ignoring their sexual health needs and desires [10, 11, 12]. This can leave them without access to appropriate education, resources, and sexual health services. Additionally, it hinders them from openly discussing their sexuality, sexual concerns, and needs with others, including healthcare providers [13, 14].

Research on sexual health primarily focused on younger age groups or explored sexual health in disability or aging independently. However, research has not specifically addressed sexual health among middle‐aged and older disabled adults. Previous research has demonstrated that both middle‐aged and older adults, as well as people with disabilities, are “sexual beings” and have needs for sexual health services [5, 15, 16]. A cross‐sectional study by Gott et al. reported that 80% of adults aged 50–90 years in the United Kingdom are still sexually active [17]. Additionally, the prevalence of sexually transmitted infections (STIs) among UK adults aged 45 years and over has almost doubled in the last decade, increasing from 9% to 16% [18]. Similarly, findings from the National Surveys of Sexual Attitudes and Lifestyles‐3 (Natsal‐3), a quantitative study exploring sexual behaviors and outcomes in Britain, indicate that young adults with disabilities are sexually active and have sexual health needs in the same way as their nondisabled counterparts [16, 19, 20].

There is a need for further research to better understand sexual health among middle‐aged and older disabled adults, as existing services do not adequately serve them [21, 22, 23]. This study aims to fill this evidence gap, recognizing that approximately 15.0 million people in the United Kingdom have a disability, with almost half of them aged 45 years and over [24]. This study quantitatively investigated sexual behaviors and outcomes among middle‐aged and older disabled adults (aged 45–74) compared to those without disabilities in Britain.

## 2 Methods

### 2.1 Data Source and Sampling Methods

We performed a secondary quantitative analysis using data from Natsal‐3, which used a multistage probability sample design with clustering and stratification to obtain a sample of 15,162 respondents, aged 16–74 in Britain [25]. Natsal‐3 is notable for its expanded coverage in terms of age. The next in the Natsal series, Natsal‐4, will only sample individuals aged 16–59, and so with Natsal‐3 there is an especial opportunity to investigate the farther end of the life cycle.

The Natsal project is run by a multi‐disciplinary team of researchers from University College London (UCL), the London School of Hygiene & Tropical Medicine (LSHTM), the University of Glasgow, University of Cambridge, Örebro University Hospital, and NatCen Social Research (NatCen). The study used primary sampling units as postcode units and randomly chose one eligible adult from each household. It applied weighting to account for unequal probabilities of selection and nonresponse.

The Natsal‐3 team conducted fieldwork from 2010 to 2012. Researchers sought oral consent from participants and administered the questionnaire through a face‐to‐face computer‐assisted personal interview (CAPI). For more sensitive questions, like sexual health components, researchers employed self‐completed computer‐assisted self‐interview techniques. Full details of the methodology are available online [25, 26].

### 2.2 Study Population and Selection Criteria

The population of interest for our analysis is disabled adults aged 45–74 [27, 28]. We categorized disabled individuals as being of limiting disability status if Natsal‐3 participants responded ‘yes’ to both questions: “Do you have any long‐standing illness, disability or infirmity?” and “Does this limit your activities in any way?” This categorization aligns with the definition used in the UK Equality Act [29] and the UN Convention on the Rights of Persons with Disabilities [3].

### 2.3 Study Variables

We recoded all included variables for this secondary analysis and categorized those that fell into the ‘not applicable’ or ‘not answered’ as missing [30]. Additional information on the reclassification of variables is available in Tables A1, B1, C1.

#### 2.3.1 Variables on Participant Characteristics (Table A1)

We included the Natsal‐3 sociodemographic characteristics (age, academic qualifications, National Statistics Socio‐Economic Classification (NS‐SEC) code, ethnicity, Index of Multiple Deprivation (IMD) quantile, general health and current depression factors) for the analysis. We constructed variables on education, ethnicity, and socioeconomic status [31, 32].

Respondents were surveyed about their education level in the CAPI via being shown a card with 16 items, including “degree level qualification,” “A‐level,” “AS level,” and so forth. This was accompanied by a question: “Have you passed any exams or got any qualifications on this card?” People who had at least one of the exams or qualifications listed on the card would be prompted to choose the “highest qualification” among them that they possessed. The choices were then grouped into three categories: “No academic qualifications,” “Academic qualifications typically gained at age 16,” and “Studying for/attained further academic qualifications.”

Similarly, we displayed a card with a list of ethnic groups to participants. The major groups to which participants could identify themselves as belonging to included “White,” “Mixed,” “Asian or Asian British,” “Black or British Black,” and “Chinese or other.” Due to the small number of respondents belonging to the mixed and Chinese or other groups, these two categories were merged for the analysis.

The sole open‐ended questions within the survey were on the main jobs of respondents and their partners', if any. Participants could provide typed descriptions of these jobs, and Natsal‐3 coders classifying them using the Standard Occupational Classification. Together with another card‐based question on employment status, which offered options such as “In paid employment or self‐employed (or temporarily away),” “Looking for paid work or a Government training scheme (unemployed),” and “Retired from paid work,” the NS‐SEC for respondents could be derived [31]. The four‐group variant of the NS‐SEC included in the original dataset was used for this project. The following are the four groups: managerial and professional jobs, intermediate occupations, routine jobs and the long‐term unemployed. In addition, we coded them “Full‐time students” to fully cover the adult population.

Aside from using the NS‐SEC as a social class measure, we selected an IMD quintile variable to indicate respondents' broader socioeconomic determinants. Adjusted based on a method by Payne and Abel [33] to enable comparisons between England, Scotland and Wales, areas in the first quintile of the IMD are regarded as being the least deprived, while those in the fifth quintile are designated as being the most.

#### 2.3.2 Independent Variables

The primary independent variables in this study are sex, limiting disability status, and age group. Age was stratified into three categories: 45–54, 55–64, and 65–74 years. We employed age groupings consistent with prior Natsal‐3 analyses [5] to maintain comparability and ensure sufficient sample sizes within each category.

#### 2.3.3 Dependent Variables (Tables B1 and C1)

Our primary dependent variables for this study were sexual health‐related behavior (frequency of sex within last four weeks, types of sexual act performed, relationship status whether the participant reported in a steady relationship or had a current partner, same and heterosexual sex partners, usage of condom in heterosexual vaginal/anal sex, ever attend a sexual health clinic, and sought help/advice for sex life in past year) and outcomes (ever experienced coerced sex attempts, ever experienced coerced sex, ever diagnosed with a STI, low sexual function, sexual satisfaction, distressed by/worried about sex, sex life affected by health last year, and ever paid money for sex). The Netsal‐3 assessed sexual function [34] using the Natsal Sexual Function Measure (Natsal‐SF). This instrument allows researchers to examine the psychological aspects of sex and aspects of sexual dysfunctions, for example, vaginal dryness and erection difficulties.

### 2.4 Statistical Analysis

We conducted all statistical analyses using Stata SE 17.0, with Stata's complex survey functions. We examined associations between limiting disability status and participants' demographic characteristics, sexual health‐related behaviors and outcomes using bivariate analyses. We considered *p* < 0.05 to indicate statistical significance, and where relevant, we report 95% confidence intervals (CIs).

We conducted multiple logistic regression to obtain adjusted odds ratios by controlling for potential confounding variables, specifically the age group, sex, education level, and social class categorized by the NS‐SEC [7, 35, 36, 37, 38]. Further multiple logistic regression models assessed how the odds for sexual behaviors and outcomes differed by limiting disability status for men and women and different age groups.

### 2.5 Ethics Statement

The Oxfordshire Research Ethics Committee A granted ethical approval for Natsal‐3 (reference: 09/H0604/27).

## 3 Results

### 3.1 Demographic Characteristics

After applying sample weights, the sample for our analysis included 7082 respondents aged 45–74. Of those, 3469 were men and 3613 were women; there were 1906 respondents with limiting disability status and 5176 with no reported limiting disability (Table 1).

**TABLE 1:** Variation in reporting of sociodemographic characteristics and health‐related factors by limiting disability status in Britain, stratified by sex, 2010–2012, *n* = 7082.

	Men, *n* = 3469; weighted count (%)^a^		*p*	Women, *n* = 3613; weighted count (%)^a^		*p*
	No reported limiting disability, *n* = 2583	Reported limiting disability, *n* = 886		No reported limiting disability, *n* = 2593	Reported limiting disability, *n* = 1020	
Age			< 0.001*			0.001*
45–54	1142 (44.2)	271 (30.6)		1091 (42.1)	352 (34.5)	
55–64	850 (32.9)	347 (39.1)		871 (33.6)	363 (35.6)	
65–74	592 (22.9)	268 (30.3)		630 (24.3)	304 (29.8)	
Academic qualifications			< 0.001*			0.001*
No academic qualifications	709 (27.8)	417 (47.6)		778 (30.8)	432 (43.1)	
Qualifications typically obtained at age 16	797 (31.2)	225 (25.7)		886 (35.1)	350 (34.9)	
Studying for/further academic qualifications	1046 (41.0)	233 (26.7)		865 (34.2)	221 (22.0)	
NS‐SEC code			< 0.001*			< 0.001*
Managerial/professional	1098 (42.8)	233 (26.3)		877 (34.1)	201 (19.8)	
Intermediate	499 (19.5)	159 (18.0)		590 (22.9)	147 (14.4)	
Semi‐routine/routine	782 (30.5)	256 (29.0)		647 (25.1)	265 (26.1)	
No job	168 (6.6)	232 (26.3)		444 (17.2)	401 (39.5)	
Full‐time student	15 (0.6)	3 (0.4)		17 (0.7)	1 (0.1)	
Ethnicity			0.30			0.57
White	2369 (92.3)	832 (94.3)		2375 (91.8)	942 (92.6)	
Asian/Asian British	100 (3.9)	26 (2.9)		78 (3.0)	31 (3.1)	
Black/Black British	66 (2.6)	11 (1.3)		86 (3.3)	23 (2.2)	
Mixed/Chinese/other	31 (1.2)	13 (1.5)		48 (1.8)	22 (2.1)	
IMD quintile			< 0.001*			< 0.001*
1 (least deprived)	695 (26.9)	114 (12.8)		671 (25.9)	172 (16.8)	
2	642 (24.9)	184 (20.8)		636 (24.5)	175 (17.2)	
3	493 (19.1)	181 (20.4)		489 (18.9)	206 (20.2)	
4	409 (15.8)	177 (20.0)		426 (16.4)	223 (21.9)	
5 (most deprived)	344 (13.3)	230 (26.0)		370 (14.3)	244 (23.9)	
General health			< 0.001*			< 0.001*
Fair to very good	2569 (99.4)	654 (74.1)		2577 (99.4)	747 (73.3)	
Very bad to bad	15 (0.6)	229 (25.9)		16 (0.6)	273 (26.7)	
Current depression^b^			< 0.001*			< 0.001*
Yes	122 (5.0)	182 (22.3)		156 (6.2)	207 (21.7)	
No	2337 (95.0)	632 (77.7)		2356 (93.8)	747 (78.3)	

Abbreviations: IMD, Index of Multiple Deprivation; NS‐SEC, National Statistics Socio‐Economic Classification.

^a^ Valid percentages.

^b^ As screened for via Patient Health Questionnaire‐2. Those who scored ≥ 3 were screened as positive (yes) for current depression.

* Statistically significant at *p* < 0.05.

### 3.2 Sexual Health‐Related Behaviors

Table 2 compares sexual health‐related behaviors by limiting disability status and sex. Overall, in recent sexual activities, people reporting limiting disability status, both men and women, engaged less compared to nondisabled people, including a number of occasions of sex (*p* < 0.001), vaginal sex (*p* < 0.001), oral sex (*p* < 0.001) and genital contact (*p* = 0.009 for men, *p* = 0.001 for women). Fewer disabled than nondisabled men masturbated in the past week (*p* = 0.003), and fewer disabled men (*p* < 0.001) and women (*p* = 0.001) reported a steady relationship. There was some evidence that men with disabilities were less likely to have used condoms on every occasion of vaginal or heterosexual anal sex they had in the last year (*p* = 0.03). No difference was found between disabled and nondisabled participants for ever attending a sexual health clinic (*p* = 0.94 for men, *p* = 0.12 for women), but disabled men were more likely to have sought help or advice for their sex life from any source in the past year (*p* < 0.001).

**TABLE 2:** Variation in reporting of sexual health‐related behaviors by limiting disability status in Britain, stratified by sex, 2010–2012, *n* = 7082.

	Men, *n* = 3469; weighted count (%)^a^		*p*	Women, *n* = 3613; weighted count (%)^a^		*p*
	No reported limiting disability, *n* = 2583	Reported limiting disability, *n* = 886		No reported limiting disability, *n* = 2593	Reported limiting disability, *n* = 1020	
No. of occasions of sex in last 4 weeks			< 0.001*			< 0.001*
0	833 (35.2)	425 (55.2)		1172 (48.8)	590 (65.1)	
1–2	639 (26.9)	145 (18.8)		519 (21.6)	133 (14.7)	
3–4	385 (16.2)	106 (13.8)		363 (15.1)	79 (8.7)	
5+	513 (21.7)	94 (12.2)		349 (14.5)	104 (11.5)	
Had vaginal sex within last 4 weeks	1534 (62.1)	336 (40.8)	< 0.001*	1247 (49.9)	330 (34.5)	< 0.001*
Had anal sex within the last 4 weeks	86 (3.5)	32 (3.9)	0.68	55 (2.1)	21 (2.1)	0.99
Had oral sex within last 4 weeks	940 (38.1)	213 (26.0)	< 0.001*	647 (26.1)	166 (17.5)	< 0.001*
Had genital contact without intercourse within last 4 weeks	944 (38.2)	255 (31.2)	0.009*	759 (30.4)	220 (23.1)	0.001*
No. of occasions of masturbation in the last week			0.003*			0.32
0	1575 (64.6)	577 (73.0)		2205 (89.3)	858 (91.4)	
1–2	548 (22.5)	153 (19.3)		226 (9.2)	66 (7.0)	
3–4	214 (8.8)	39 (5.0)		30 (1.2)	11 (1.2)	
5+	102 (4.2)	22 (2.8)		8 (0.3)	5 (0.5)	
Relationship status			< 0.001*			0.001*
Married/civil partnership	1828 (72.1)	524 (62.3)		1676 (65.9)	599 (60.4)	
Living with partner	229 (9.0)	67 (7.9)		184 (7.2)	56 (5.6)	
Steady but not living together	136 (5.4)	38 (4.5)		138 (5.4)	55 (5.5)	
Not in a steady relationship	342 (13.5)	212 (25.3)		545 (21.4)	283 (28.5)	
No. of heterosexual sex partners in the last 4 weeks			< 0.001*			< 0.001*
0	895 (36.5)	467 (57.2)		1212 (49.0)	616 (65.6)	
1	1506 (61.4)	342 (41.8)		1254 (50.7)	322 (34.3)	
2+	51 (2.1)	8 (0.9)		6 (0.2)	1 (0.1)	
Had at least 1 same‐sex sex partner within the last 4 weeks	23 (0.9)	13 (1.5)	0.28	11 (0.4)	3 (0.3)	0.53
Always had heterosexual vaginal/anal sex with a condom in last year (*n* = 4614)	163 (8.3)	24 (4.8)	0.03	114 (6.9)	40 (8.1)	0.44
Ever attended a sexual health clinic	353 (14.6)	115 (14.5)	0.94	260 (10.6)	119 (12.7)	0.12
Sought help/advice for sex life in past year	247 (10.1)	137 (17.1)	< 0.001*	199 (8.0)	93 (9.8)	0.15

^a^ Valid percentages.

* Statistically significant at *p* < 0.05.

### 3.3 Sexual Health Outcomes

Table 3 shows reported sexual health outcomes by limiting disability status and sex. A higher proportion of disabled men (*p* = 0.02) and women (*p* < 0.001) had experienced coerced sex attempts compared to their nondisabled counterparts. Additionally, a greater number of disabled women reported ever experiencing coerced sex (*p* < 0.001).

**TABLE 3:** Variation in reporting of sexual health outcomes by limiting disability status in Britain, stratified by sex, 2010–2012, *n* = 7082.

	Men, *n* = 3469; weighted count (%)^a^		*p*	Women, *n* = 3613; weighted count (%)^a^		*p*
	No reported limiting disability	Reported limiting disability		No reported limiting disability	Reported limiting disability	
Ever experienced coerced sex attempts^b^	116 (4.7)	62 (7.5)	0.02	440 (17.7)	233 (24.6)	< 0.001*
Ever experienced coerced sex^c^	31 (1.2)	14 (1.6)	0.12	200 (7.7)	130 (12.7)	< 0.001*
Ever diagnosed with an STI	294 (12.1)	110 (13.8)	0.34	933 (37.7)	344 (36.5)	0.57
Low sexual function according to Natsal‐SF (*n* = 4846)	392 (19.8)	194 (35.6)	< 0.001*	396 (22.4)	173 (31.4)	< 0.001*
Dissatisfied with sex life in last year	427 (17.5)	188 (23.6)	0.006*	327 (13.2)	183 (19.5)	< 0.001*
Distressed by/worried about sex life in last year	238 (9.7)	143 (18.0)	< 0.001*	206 (8.3)	140 (14.9)	< 0.001*
Sex life affected by health in last year	381 (15.8)	362 (45.8)	< 0.001*	263 (10.7)	352 (37.7)	< 0.001*
Ever paid money for sex with a man/women	289 (11.7)	105 (12.7)	0.90	2 (0.1)	5 (0.5)	0.16

Abbreviation: Natsal Sexual Function Measure.

^a^ Valid percentages.

^b^ Anyone tried to make you have sex with them against your will (aged 13+).

^c^ Anyone actually made you have sex with them against your will (aged 13+).

* Statistically significant at *p* < 0.05.

Statistically, more disabled adults had low sexual function, including both men and women (*p* < 0.001). There was a correlation between having a disability and sexual dissatisfaction for both disabled men (*p* = 0.006) and disabled women (*p* < 0.001). Distress or worry about one's sex life was almost two times more common (*p* < 0.001) among disabled participants compared to nondisabled participants. A higher proportion of disabled people asserted that their health affected their sex lives in the last year (*p* < 0.001).

### 3.4 Multiple Logistic Regression

#### 3.4.1 Limiting Disability Status as an Independent Variable

Table 4 compares the odds of sexual health behaviors and outcomes among disabled and nondisabled individuals between the ages of 45–74. These disabled adults were less likely to engage in sexual activities in the last 4 weeks (aOR: 0.60, CI: 0.51–0.71) and less likely to be in a steady relationship (aOR: 0.69, CI: 0.59–0.80). The odds of disabled adults ever attending sexual health clinics were relatively high (aOR: 1.35, CI: 1.08–1.69). Moreover, they were more likely to have sought help for their sex life in the last year (aOR: 1.73, CI: 1.38–2.17) compared to nondisabled people in the same age category (45–74 years of age).

**TABLE 4:** Results of logistic regression models comparing sexual health‐related behaviors and outcomes by limiting disability status in Britain, 2010–2012, *n* = 7082.

Sexual health‐related behavior and outcome	Adjusted^a^ OR (95% CI)
*Behavior*	
Sexual activity^b^ within last 4 weeks	0.60 (0.51–0.71)
Always had heterosexual vaginal/anal sex with a condom in last year	0.87 (0.62–1.22)
In a steady relationship	0.69 (0.59–0.80)
Ever attended a sexual health clinic	1.35 (1.08–1.69)
Sought help/advice for sex life in past year	1.73 (1.38–2.17)
*Outcome*	
Ever experienced coerced sex attempts^c^	1.83 (1.48–2.27)
Ever experienced coerced sex^d^	1.64 (1.33–2.01)
Ever diagnosed with an STI	1.23 (1.04–1.46)
Low sexual function according to Natsal‐SF	1.90 (1.56–2.32)
Dissatisfied with sex life in last year	1.68 (1.40–2.03)
Distressed by/worried about sex life in last year	2.00 (1.59–2.50)
Sex life affected by health in last year	5.08 (4.27–6.05)
Ever paid money for sex with a man/woman	0.79 (0.57–1.10)

*Note*: Nondisabled participants as a reference group.

^a^ Adjusted for age group, sex, academic qualifications, and NS‐SEC.

^b^ Sexual activity includes reported vaginal, anal, or oral sex and genital contact without intercourse and masturbation.

^c^ Anyone tried to make you have sex with them against your will (aged 13+).

^d^ Anyone actually made you have sex with them against your will (aged 13+).

Sexual outcomes were poorer for disabled people aged 45–74, including higher odds of ever experiencing coerced sex attempts (aOR: 1.83, CI: 1.48–2.27) and coerced sex (aOR: 1.64, CI: 1.33–2.01). Also, among people who reported sex within the last year, disabled people aged 45–74 were more likely to report low sexual function (aOR: 1.90, CI: 1.56–2.32). Disabled participants in this study had greater odds of being dissatisfied and distressed/worried about their sex life in the last year. Furthermore, disabled participants experienced significantly higher odds of their sex life being affected by their health in the last year (aOR: 5.08, CI: 4.27–6.05).

Table 5 lays out the results for the logistic regression models on behaviors and outcomes stratified by sex compared to nondisabled counterparts; it indicates similar patterns as those in Table 4. Disabled men were less likely to have sex with a condom in the last year (aOR: 0.52, CI: 0.30–0.89), and they were more likely to ever be diagnosed with an STI (aOR: 1.38, CI: 1.003–1.890). Disabled women showed higher odds of attending sexual health clinics at any point (aOR: 1.44, CI: 1.08–1.93). Table D1 presents the odds ratios for behaviors and outcomes among disabled participants, stratified by age group.

**TABLE 5:** Results of logistic regression models comparing sexual health‐related behaviors and outcomes by limiting disability status in Britain, stratified by sex, 2010–2012, *n* = 7082.

	Men, *n* = 3469	Women, *n* = 3613
Sexual health‐related behavior/outcome	Adjusted^a^ OR (95% CI)	Adjusted^a^ OR (95% CI)
*Behavior*		
Sexual activity^b^ within last 4 weeks	0.60 (0.45–0.80)	0.61 (0.50–0.76)
Always had heterosexual vaginal/anal sex with a condom in last year	0.52 (0.30–0.89)	1.32 (0.84–2.05)
In a steady relationship	0.60 (0.47–0.77)	0.76 (0.63–0.92)
Ever attended a sexual health clinic	1.29 (0.92–1.80)	1.44 (1.08–1.93)
Sought help/advice for sex life in past year	1.94 (1.41–2.68)	1.49 (1.07–2.07)
*Outcome*		
Ever experienced coerced sex attempts^c^	2.13 (1.32–3.42)	1.74 (1.39–2.19)
Ever experienced coerced sex^d^	1.10 (0.73–1.63)	1.90 (1.49–2.44)
Ever diagnosed with an STI	1.38 (1.003–1.89)	1.17 (0.96–1.43)
Low sexual function according to Natsal‐SF	2.21 (1.63–2.99)	1.64 (1.25–2.15)
Dissatisfied with sex life in last year	1.55 (1.17–2.06)	1.82 (1.43–2.32)
Distressed by/worried about sex life in last year	1.94 (1.39–2.71)	1.99 (1.46–2.72)
Sex life affected by health in last year	4.52 (3.48–5.87)	5.72 (4.47–7.31)
Ever paid money for sex with a man/woman	0.83 (0.60–1.15)	0.05 (0.004–0.59)

*Note*: Nondisabled participants as a reference group.

^a^ Adjusted for age group, academic qualifications, and NS‐SEC.

^b^ Sexual activity includes reported vaginal, anal, or oral sex and genital contact without intercourse and masturbation.

^c^ Anyone tried to make you have sex with them against your will (aged 13+).

^d^ Anyone actually made you have sex with them against your will (aged 13+).

## 4 Discussion

In this secondary analysis of Natsal‐3, we explored sexual behavior and outcomes among disabled adults aged 45 to 74 in Britain. Overall, they engaged less in sexual activities and demonstrated poorer sexual health outcomes. Notably, they were more likely to seek help and to have exhibited increased vulnerability as having experienced coerced sex.

The findings underscore the complexity of sexual health among middle‐aged and older disabled individuals. While our analysis revealed that disabled adults were less sexually active, this does not imply that all disabled adults aged 45–74 are sexually inactive or have no sexual health needs. In fact, our results indicate that they are more likely to seek assistance with their sexual health concerns and attend sexual health clinics compared to nondisabled counterparts.

This highlights the importance of addressing the unique sexual health challenges faced by middle‐aged and older disabled adults. Previous literature has identified specific needs among young disabled people, including difficulties in finding suitable partners [20], worries or dissatisfaction with sexual activity [16], and sexual dysfunction associated with physical or psychological impairments [39, 40]. Our study contributes to this by suggesting that these specific sexual health needs may persist beyond young adulthood and into middle age and older adulthood. However, it is concerning that only a few clinics offer specialized services for disabled individuals in this age group [21, 41], and there remains limited evidence on whether middle‐aged and older disabled adults receive the necessary support they need [5, 42, 43]. To bridge these gaps in care, healthcare providers could initiate open discussions about sexual health with their middle‐aged and older patients with disabilities. Additionally, sexual health clinics could enhance their facilities and services to be more inclusive and accommodating for them.

Another significant insight gained from this study is that disabled adults aged 45–74 had higher odds of experiencing coerced sex attempts and coerced sex at some point in their lives. Prior research has extensively documented the heightened risk of sexual assault among individuals with disabilities, along with the prevalence of such incidents among middle‐aged and older adults [44, 45, 46]. Moreover, existing literature suggests that experiences of sexual assault at a young age may have an enduring impact on sexual health behavior, outcomes, and relationships in their later life [47, 48]. The question of whether the experience of sexual assault has a long‐term effect on the sexual health of individuals into middle age and older years warrants exploration in future research.

### 4.1 Strengths

This study brings additional insights into sexual health and disability research among adults aged 45–74, using a nationally representative sample in Britain. Natsal‐3 provides a unique opportunity to examine these questions as the following Natsal‐4 excludes this age group.

### 4.2 Limitations

First, this is a cross‐sectional study, so we cannot infer causal relationships. Second, the study did not sample institutions or other locations where adults 45–74 live, likely under‐representing this group [25]. Third, the study did not distinguish those with lifetime disabilities from those who became disabled by aging. Finally, the sexual behaviors and outcomes of the British disabled and nondisabled subpopulations may have changed since the end of Natsal‐3 data collection; although the data are no longer current, the continued lack of attention on sexual health issues among disabled adults age 45–74, despite the aging population in Britain, suggests that our analysis is still relevant today.

## 5 Conclusion

Disabled adults aged 45–74 in Britain had lower sexual activities, yet reported negative sexual health outcomes. Their increased health‐seeking behaviors and their vulnerability highlight the necessity for tailored sexual health services, extending into middle age and older adulthood. Future research could explore how sexual health services can effectively address the needs of those disabled in this age group.

## Conflicts of Interest

The authors declare no conflicts of interest.

## Supporting information


**Data S1:** Supporting Information.

## Appendix A See Table A1

**TABLE A1:** Variables on participant characteristics.

Variable	Recoded variables in this study	How variables was asked in Natsal‐3
Academic qualifications	People who had at least one of the exams or qualifications listed on the card would be prompted to choose the “highest qualification” among them that they possessed: No academic qualifications Qualifications typically obtained at age 16 Studying for/further academic qualifications	Participants were asked: At what age did you complete your continuous full‐time education? If you had a “gap” year between school and university or college please include it as continuous. Have you passed any exams or got any of the qualifications? Yes No, none IF Yes THEN Please read down the list and tell me the highest qualification that you have, that is, the first one you come to. INTERVIEWER: Code one only. Degree level qualification A‐levels AS level SLC higher grade, etc. O‐level, 1975 or earlier O‐level, after 1975 A–C O‐level, after 1975 D–E GCSE grades A*–C GCSE grades D–G CSE grade 1, etc. CSE grades 2–5, etc. CSE ungraded SLC lower SUPE lower or ordinary School certificate Foreign qualification
Socioeconomic class (NS‐SEC code)	Managerial/professional Intermediate Semi‐routine/routine No job Full‐time student	Questions asked here were informed by the International Standard Occupation codes (SOC). For more information, see the International SOC: http://www.ons.gov.uk/ons/guide‐method/classifications/archived‐standard‐classifications/standard‐occupational‐classification‐2000/index.html. Following the Natsal‐3 groupings, participants responses were classified as Managerial and professional occupations Intermediate occupations Semi‐routine/routine occupations Never worked/no job of 10+ h/week/not in the last 10 years Student in full‐time education
Ethnicity	Due to the small number of respondents belonging to the mixed and Chinese or other groups, these two categories were merged for the analysis: White Asian/Asian British Black/Black British Mixed/Chinese/other	Participants were asked about which ethnic group they belong to. They could choose: White British Irish Any Other White background Mixed 4 White and Black Caribbean 5 White and Black African 6 White and Asian 7 Any other mixed background Asian or Asian British 8 Indian 9 Pakistani 10 Bangladeshi 11 Any other Asian background Black or British Black 12 Caribbean 13 African 14 Any other black background Asian or Asian British 15 Chinese 16 Any other
Quantile of index of multiple deprivation	1 (Least deprived) 5 (Most deprived)	Postcodes were used to obtain IMD scores. The adjusted IMD score was generated using a method by Payne and Abel. The individual country IMD scores combined with the coefficient and residual values from a linear regression of income and employment on the overall IMD score for each country. The combined scores were generated using the most up‐to‐date scores for each country at the time. These were IMD 2010 for England, IMD 2011 for Wales, and IMD 2009 for Scotland. Respondents were classified: (Least deprived) (Most deprived)
General health	In this study, this general health variable has been dichotomized so that “fair” to “very good” self‐reported health is grouped together and “very bad” to “bad” health is considered poor. Fair to very good Very bad to bad	Respondents were asked: “How is your health in general?” Very good Good Fair Bad Very bad
Current depression	To further gauge wellbeing, Natsal‐3 also checked for participants' mood using the validated Patient Health Questionnaire‐2 (PHQ‐2) measure, thus screening for depressive symptoms. This consisted of two questions wherein respondents should have selected the frequency with which they had been perturbed by “little interest/pleasure in doing things” and/or “feeling down, depressed or hopeless” over the past 2 weeks. A score would be generated according to their answers. Those who scored 3 or above are seen as screening positive for current depression: Yes No	Respondents were asked: “In the last 12 months, that is since (date 12 months ago), have you received treatment from a health professional for any of the medical conditions listed on this card?” Yes No IF Yes THEN “Which ones in the last year?” Back ache lasting for 3 months or longer Any other muscle or bone disease lasting for 3 months or longer Depression Any other mental health condition Any other neurological condition, apart from Parkinson's disease and epilepsy Cancer Any thyroid condition Any (ovarian/testicular) or pituitary condition [WOMEN ONLY] Polycystic ovaries [WOMEN ONLY] I have received IVF or other fertility treatment Respondents could select and record as many conditions as needed. If depression (3) was indicated as one the those treated in the past year then the respondent was classified having “mentioned” the condition

## Appendix B See Table B1

**TABLE B1:** Variables on sexual health‐related behavior.

Variable	Recoded variables in this study	How variables was asked in Natsal‐3
Had vaginal sex within last 4 weeks	New binary variables were generated for whether participants had oral sex, anal sex, or genital contact within the last 4 weeks. For this project, respondents were only viewed as having engaged in vaginal sex, oral sex, anal sex or genital contact if they had chosen “In the last 7 days” or “Between 7 days and 4 weeks ago” for any of the corresponding question(s)	Respondents were asked: “When, if ever, was the last occasion you had vaginal sexual intercourse with a (woman/man)?**”** In the last 7 days Between 7 days and 4 weeks ago Between 4 weeks and 6 months ago Between 6 months and 1 year ago Between 1 year and 5 years ago Longer than 5 years ago Never had vaginal intercourse
Had anal sex within last 4 weeks		Respondents were asked: “When, if ever, was the last occasion you had anal sex with a (woman/man)?**”** In the last 7 days Between 7 days and 4 weeks ago Between 4 weeks and 6 months ago Between 6 months and 1 year ago Between 1 year and 5 years ago Longer than 5 years ago Never had anal sex
Had oral sex within last 4 weeks		Respondents were asked: **“**When, if ever, was the last occasion you had oral sex with a (woman/man)—by (her/him) to you, that is (her/his) mouth on your genital area?” In the last 7 days Between 7 days and 4 weeks ago Between 4 weeks and 6 months ago Between 6 months and 1 year ago Between 1 year and 5 years ago Longer than 5 years ago Never had oral sex—(woman/man) to me
Had genital contact without intercourse within last 4 weeks		Respondents were asked: **“**When, if ever, was the last occasion you had genital contact without intercourse with a (woman/man)?” In the last 7 days Between 7 days and 4 weeks ago Between 4 weeks and 6 months ago Between 6 months and 1 year ago Between 1 year and 5 years ago Longer than 5 years ago Never had genital contact (without intercourse as well)
No. of occasions of sex in last 4 weeks	0 1–2 3–4 5+	Respondents were asked: **“**On how many occasions in the last 4 weeks have you had sex with a (woman/man)?” **“**Type in the number of occasions in the last 4 weeks”
No. of occasions of masturbation in last week	0 1–2 3–4 5+	Respondents were asked: **“**In the last 7 days, how many times have you masturbated?” **“**Type in the number of occasions in the last 4 weeks”
Relationship status	Married/civil partnership Living with partner Steady but not living together Not in steady relationship	At present are you… Married and living with your (husband/wife) In a registered same‐sex civil partnership and living with your partner Living with a partner, as a couple (not married or in a civil partnership) In a steady relationship, but not living together None of the above Married or civil partnership if their response was 1 or 2 Living with partner if their response was 3 In steady relationship, not cohabitating if their response was 4 No steady relationship if their response was 5
No. of heterosexual sex partners in last 4 weeks	0 1 2+	Respondents were asked: **“**How many (women/men) have you had sex with in last 4 weeks?” **“**Type in the number of occasion in the last 4 weeks”
Had at least 1 same‐sex sex partner within last 4 weeks	The number of people reporting any same‐sex partners at all for the same period was small, and therefore the equivalent variable has been modified Yes No	Respondents were asked: **“**How many (women/men) have you had sex with in last 4 weeks?” **“**Type in the number of occasion in the last 4 weeks”
Always had heterosexual vaginal/anal sex with a condom in last year	Not always with a condom Always with a condom	Respondents were asked: **“**In the last year have you ever had vaginal (or anal) intercourse with a (woman/man) without using a condom?” 1. Yes (have had intercourse without a condom in the past year) 2. No (have used a condom on all occasions of vaginal (or anal) intercourse in the past year)
Ever attended a sexual health clinic	Yes No	Respondents were asked: **“**Have you ever attended sexual health clinic (GUM clinic)?” 1. Yes 2. No
Sought help/advice for sex life in past year	Yes No	Respondents were asked: **“**Have you ever sought help or advice regarding your sex life from the any of following sources in the last year?” Family member/friend Information and support sites on the Internet Self‐help books/information leaflets Self‐help group Helpline GP/family doctor Sexual health/GUM/STI clinic Psychiatrist or psychologist Relationship counselor Other type of clinic or doctor Have not sought any help

## Appendix C See Table C1

**TABLE C1:** Variables on sexual health outcome.

Variable	Recoded variables in this study	How variables was asked in Natsal‐3
Ever experienced coerced sex attempts (aged 13+)	A binary variant of the corresponding response variable was applied in this project, regarding “Don't know” answers as being missing data and including individuals without any lifetime partners within the “No” group instead of excluding them. Yes No	Participants were asked: “Since the age of 13, has anyone tried to make you have sex with them, against your will?” Yes No Don't know
Ever experienced coerced sex (aged 13+)	A binary variant of the corresponding response variable was applied in this project, regarding “Don't know” answers as being missing data and including individuals without any lifetime partners within the “No” group instead of excluding them: Yes No	Participants were asked: “And since the age of 13, has anyone actually made you have sex with them, against your will?” Yes No Don't know
Ever diagnosed with an STI	Recollection of any STI diagnosis ever was recorded as a positive response in the relevant dichotomous variable from the original dataset, even if participants could not remember exactly which infection it was: Yes No	Participants were asked: Have you ever been told by a doctor or other healthcare professional that you had any of the following? Please select any that you have had, even if not transmitted by sex (women only: Thrush). If more than one, press the space bar between each number. If you have not had any, please select “none of these” at the bottom of the list. Chlamydia Gonorrhea Genital warts (venereal warts) Syphilis Trichomonas vaginalis (Trich, TV) Herpes (genital herpes) Pubic lice/crabs Hepatitis B (Men only): NSU (Non Specific Urethritis), NGU (Non Gonococcal Urethritis) (Men only): Epididymitis (Women only): Pelvic inflammatory disease (PID, salpingitis) (Women only): Vaginal thrush (candida, yeast infection) (Women only): Bacterial vaginosis Yes, but can't remember which None of these
Low sexual function according to Natsal‐SF	Generated Natsal‐SF scores were categorized into “normal” and “low” levels. People with Natsal‐SF scores in the lowest quintile for their sex were classified as having low sexual function.	Participants responded to the 17‐item Natsal‐SF scale, which combines measures of issues related to physical function, mental distress, and satisfaction. While some were asked all 17 items, those whom have never been in a relationship or have not been sexually active in the past year answered only those which applied and modeling techniques were used to estimate the missing answers.
Dissatisfied with sex life	Yes No	Participants were asked: I feel satisfied with my sex life: Agree strongly Agree Neither agree nor disagree Disagree Disagree strongly Participants were classified as “dissatisfied” with their sex life if their response was 4 or 5.
Distressed by/worried about sex life	Yes No	Participants were asked: I feel distressed or worried about my sex life: Agree strongly Agree Neither agree nor disagree Participants were classified as “distressed” or “worried” about their sex life if their response was 1 or 2
Sex life affected by health in last year	Yes No	Respondent were asked: “In the last year, have you had any health condition or disability that you feel has affected your sexual activity or enjoyment in any way?” Yes No
Ever paid money for sex with a man/women	Yes No	Respondent were asked: “Have you ever paid money for sex with a (man/woman—opposite sex)?” Yes No

## Appendix D See Table D1

**TABLE D1:** Results of logistic regression models comparing sexual health‐related behaviors and outcomes by age group among respondents with limiting disability status in Britain, 2010–2012, *n* = 1905.

	Age 55–64	Age 65–74
Sexual health‐related behavior/outcome	Adjusted^a^ OR (95% CI)	Adjusted^a^ OR (95% CI)
*Behavior*		
Sexual activity^b^ within last 4 weeks	0.46 (0.33–0.64)	0.24 (0.17–0.35)
Always had heterosexual vaginal/anal sex with a condom in last year	1.67 (0.79–3.52)	2.63 (1.20–5.76)
In a steady relationship	1.00 (0.75–1.34)	0.96 (0.71–1.31)
Ever attended a sexual health clinic	0.55 (0.37–0.82)	0.41 (0.24–0.68)
Sought help/advice for sex life in past year	0.83 (0.54–1.26)	0.55 (0.33–0.90)
*Outcome*		
Ever experienced coerced sex attempts^c^	0.77 (0.53–1.10)	0.72 (0.48–1.10)
Ever experienced coerced sex^d^	0.66 (0.44–0.98)	0.55 (0.37–0.84)
Ever diagnosed with an STI	0.77 (0.55–1.06)	0.43 (0.29–0.64)
Low sexual function according to Natsal‐SF	0.94 (0.64–1.37)	1.08 (0.70–1.65)
Dissatisfied with sex life in last year	1.04 (0.73–1.46)	0.93 (0.65–1.34)
Distressed by/worried about sex life in last year	0.77 (0.54–1.12)	0.42 (0.27–0.66)
Sex life affected by health in last year	1.01 (0.75–1.37)	0.79 (0.59–1.07)
Ever paid money for sex with a man/woman	0.61 (0.31–1.21)	0.71 (0.36–1.42)

*Note*: Disabled participants aged 45–54 are the reference group.

^a^ Adjusted for age group, sex, academic qualifications, and NS‐SEC.

^b^ Sexual activity includes reported vaginal, anal, or oral sex and genital contact without intercourse and masturbation.

^c^ Anyone tried to make you have sex with them against your will (aged 13+).

^d^ Anyone actually made you have sex with them against your will (aged 13+).
