# Supplementary material for: Sexual Health Behaviors and Outcomes Among Middle‐Aged and Older Disabled Adults in Britain
Source: Perspect Sex Reprod Health. 2025 Sep 4;57(4):453–69. doi: 10.1111/psrh.70034 (PMC12747597; doi:10.1111/psrh.70034)
Supplement: Supplementary file 1 — Data S1: Supporting Information. [file PSRH-57-453-s001.docx]

**Bibliographies**

**Bernice Lin, MSc**

Bernice Lin, MSc is a student of the Bachelor of Medicine and Bachelor of Surgery programme at the University of Hong Kong. She received her MSc in Reproductive & Sexual Health Research from the London School of Hygiene and Tropical Medicine.

**Yoshiko Sakuma, MPH**

Yoshiko Sakuma, MPH is a Physiotherapist and Research Assistant at the London School of Hygiene and Tropical Medicine. Her physiotherapy degree is from the International University of Health and Welfare, Japan, and her MPH is from the Imperial College London, Global Health Stream. Her research focuses on people with disabilities.

**Eneyi Kpokiri, PhD**

Eneyi Kpokiri, PhD is a clinical pharmacist and Assistant Professor at the London School of Hygiene and Tropical Medicine, London. Her doctoral research focused on implementing antimicrobial stewardship programmes in low and middle-income settings. She has conducted several global crowdsourcing open calls, in-person/digital hackathons and other public engagement methods for health research.

**Junead Khan, BSc**

Junead Khan, BSc is an Associate Research Consultant at the London School of Hygiene and Tropical Medicine, and Computer Science and Biostatistics student at The University of North Carolina at Chapel Hill, studying as a Morehead-Cain Scholar.

**Huachun Zou, PhD**

He is a Professor of Epidemiology at the School of Public Health (Shenzhen), the Sun Yat-sen University, China. He received his PhD in Epidemiology from the University of Melbourne and postdoctoral training at the Kirby Institute, University of New South Wales, Sydney. His research areas include the epidemiology of HIV, HPV and other sexually transmitted infections (STIs) in at-risk populations, innovative interventions to prevent HIV/STIs, HIV/HPV-related cancer epidemiology and HPV vaccination strategy.

**Joseph D Tucker, PhD**

Joseph D. Tucker, MD, PhD, AM is an infectious diseases physician with a special interest in using crowdsourcing to improve health. Crowdsourcing has a group of diverse individuals solve a problem and then share solutions with the public. He is a Professor of Global Health at the London School of Hygiene and Tropical Medicine, a Professor of Medicine at UNC Chapel Hill, a Director of UNC Project-China, and a Chairman of SESH Global (SESH).

**Tom Shakespeare, PhD**

Trained in social and political sciences at Cambridge University, He subsequently studied for an MPhil and PhD. He has taught and researched at the Universities of Sunderland, Leeds, Newcastle and East Anglia. From 2008-2013, He was a technical officer at the World Health Organisation, Geneva, where He co-authored and co-edited the World Report on Disability (2011) and International Perspectives on Spinal Cord Injury (2014).

**Hannah Kuper, ScD**

Hannah Kuper, ScD is the co-director of the International Centre for Evidence in Disability. Her main research interest is disability in low and middle-income countries, with a particular focus on access to healthcare for people with disabilities and measuring the effectiveness of development interventions.

**Dan Wu, PhD**

Dan Wu, PhD is a professor, doctoral supervisor and academic leader of the Department of Social Medicine and Health Education, School of Public Health, Nanjing Medical University. Professor Wu Dan received a bachelor's degree in clinical medicine from Southern Medical University in 2009, a master's degree from UCL University in the United Kingdom in 2012, and a doctorate from the University of Hong Kong in 2017. The main research areas are the prevention and control of infectious diseases such as AIDS/sexual diseases and other public health services and health.
